# The Effect of Alpha-Lipoic Acid in the Treatment of Chronic Subjective Tinnitus through the Tinnitus Handicap Inventory Scores

**DOI:** 10.3390/audiolres13040043

**Published:** 2023-07-07

**Authors:** Luca Sacchetto, Daniele Monzani, Enrico Apa, Andrea Lovato, Valeria Caragli, Chiara Gherpelli, Silvia Palma, Elisabetta Genovese, Riccardo Nocini

**Affiliations:** 1 Otolaryngology-Head and Neck Surgery Department, University Hospital of Verona, 37126 Verona, Italy; 2 Otolaryngology and Audiology Unit, University of Modena and Reggio Emilia, 41100 Modena, Italy; 3 Otolaryngology Unit, Vicenza Hospital, 36100 Vicenza, Italy; 4 Audiology, Primary Care Department, AUSL Modena, 41121 Modena, Italy

**Keywords:** tinnitus, antioxidant agent, alpha-lipoic acid, hearing loss

## Abstract

Background and Objectives: Tinnitus affects millions of adults. Many therapies, including complementary and alternative medicine and tinnitus retraining therapies, have been trialed, but an effective option, particularly for chronic subjective tinnitus (CTS), is still lacking. Materials and Methods: This study investigated the effects of alpha-lipoic acid (600 mg. per day for two months) on two groups of patients using a questionnaire. One group (A) was affected by tinnitus associated with likely cochlear dysfunction and metabolic syndrome, and the other (B) was composed of subjects with acoustic nerve lesions. All the patients were asked to complete the Italian version of the tinnitus handicap inventory (THI) to determine the overall degree of perceived annoyance at the beginning and end of therapy. Pure tone averages for speech frequencies and for high frequencies were computed, and psychoacoustic pitch and loudness matches were determined for each subject before and after treatment. Results: The pure tone audiometry, pitch, loudness, and THI scores of both groups were reported. In group A, statistically significant differences were observed for the “functional” and “emotional” subscales. The total score of THI and the loudness of tinnitus were also significantly reduced. No statistically significant differences were observed in group B. Conclusions: These findings suggest a possible contribution of the antioxidant effect to the organ of Corti in subjects with metabolic syndrome and CST.

## 1. Introduction

Tinnitus can be defined as the perception of sound in the absence of an auditory stimulus [1]. It has various repercussions on the quality of life of the affected individuals, ranging from being slightly bothersome to highly incapacitating with sleep disturbances, anxiety, and depression [2]. Among the adult population, it has been estimated to have an incidence of more than 5% [3,4], and its prevalence increases with age, with any form of tinnitus being present in 10% of young adults, 14% of middle-aged adults, and 24% of older adults [5].

Most cases concern chronic subjective tinnitus (CST), whose pathophysiology has not been clearly elucidated [6]. Reported altered activity of the auditory, prefrontal, and limbic regions suggests a more systemic approach to understanding the origins of tinnitus [7]. Objective tinnitus is much rarer than subjective tinnitus and is often associated with abnormal clonic muscular contractions of the palatal or middle ear muscles and/or a variety of vascular lesions arising from the internal jugular vein or jugular bulb.

Noise exposure or aging [8] but also ototoxic drugs (gentamicin, streptomycin, and cisplatin) can induce irreversible cochlear damage with consequent hearing loss and tinnitus. On the other hand, CST can be a symptom of acoustic nerve lesions and dysfunctions such as vestibular schwannoma [9], a residual symptom of Ramsay–Hunt syndrome [10], a consequence of vascular compression of cranial nerve VIII [11], or a complaint in auditory neuropathy spectrum disorder [12].

Recent studies have focused on the possible involvement of the limbic system [13] and on the relationship between maladaptive neuroplasticity and hyperactivity in response to cochlear damage in an extended neuronal network, including the primary auditory cortex and higher-order association areas [14]. There is also evidence of an association between tinnitus and systemic arterial hypertension [15], diabetes type 2 [16,17], and hyperlipidemia [18]. Circulatory disturbance interferes with stria vascularis activity and may interrupt ionic recycling, increase the production of free radicals, and accelerate cell loss. Similarly, it is possible that these changes in cochlear microcirculation act as supporting factors in the pathophysiology of tinnitus [15]. The presence of insulin receptors, glucose transporters, and insulin signaling components in the epithelium of the cochlea as well as the stria vascularis suggests that the organ of Corti is vulnerable to impairment of glucose utilization [19].

Many clinical trials have investigated the efficacy of different drugs in the treatment of CST [20,21,22,23,24]; complementary and alternative medicine [25,26], instrumental procedures [27], and tinnitus retraining therapies [28] have been experimented with, but an effective therapy option, particularly for CTS, is still lacking [6,29].

The acknowledgment that oxidative stress is generally implicated in the pathophysiology of a variety of chronic diseases and age-related hearing loss [30,31,32] has opened up new paths in pharmacological treatment [33]. Oxidative stress can damage outer hair cells, play an important role in inducing apoptosis, and have an implication in the pathogenesis of tinnitus secondary to cochleopathies [34,35]. A recent study showed that the total oxidant status and oxidative stress index were significantly increased in tinnitus sufferers compared to healthy controls, suggesting an antioxidant enzyme imbalance between the two groups [36].

Due to its antioxidant effects, alpha-lipoic acid (ALA) has gained attention over the years in the treatment of several diseases [37], as it is considered a safe supplement without any side effects [38]. In Italy, it has also been added to the list of authorized supplements by the Ministry of Health [39].

ALA, also known as thioctic acid, is a coenzyme naturally synthesized by humans, commonly found in mitochondria, and occurring in two optical isomers: R- and S-lipoic acid [40]. Only the R-isomer is endogenously synthesized and represents an essential cofactor for several mitochondrial enzyme complexes that catalyze critical chemical reactions related to energy production. It is also involved in the regeneration of reduced glutathione, a natural protective element for the cochlea, whose depletion in the organ of Corti increases aminoglycoside ototoxicity [41,42].

In animal models, the antioxidant property of ALA results in a protective effect for the cochlea against prolonged noise exposure [43,44] and age-related and cisplatin-induced hearing loss [45,46]. Moreover, ALA acts as an anti-inflammatory agent by up-regulating the expression of nerve growth factor in experimental diabetic neuropathy [47] and counteracting the cumulative damage to Schwann cells induced by endoneurial hypoxia [48].

Based on these observations, the aim of this study was to evaluate the effect of the R-isomer form of ALA on tinnitus annoyance in two groups of patients: subjects affected by tinnitus associated with cochlear dysfunctions and metabolic syndromes and subjects with a complaint of tinnitus associated with auditory nerve pathologies. The effects were evaluated through the administration of the THI questionnaire.

## 2. Materials and Methods

### 2.1. Casuistry

The enrollment of subjects was carried out in the ENT department of the University Hospital of Modena between September 2010 and April 2012.

The inclusion criteria were steady loudness (±5 dB between test and retest) and pitch of tinnitus perceived by the subject (determined in comparison with the generated acoustic stimulation—see below) in at least two sequential controls after conventional therapies.

The exclusion criteria were: external or middle-ear diseases; fluctuating hearing loss; and a history of any major psychiatric disorders. Patients with objective tinnitus due to arteriovenous malformations or fistulas, aneurysms, vascular stenosis (particularly of the carotid arteries), and vascular tumors such as glomus jugulare tumors were excluded. Patients with palatal tremors, previously known as palatal myoclonus, were also excluded.

Fifty subjects affected by CST, unilateral or bilateral, with more severe tinnitus (the one indicated by the subject as the most bothersome) in only one ear were recruited and divided into two groups. Group A was composed of 30 subjects and included 16 males and 14 females (average age of 61.7 years, range 52–71, standard deviation 9). They were diagnosed as affected by bilateral hearing loss and metabolic syndrome (obesity, dyslipidemia, hyperglycemia, and hypertension).

The absence of auditory nerve lesions was documented by MRI. Pure tone audiometry evidenced “down-sloping” curves from middle to high frequencies (with no history of professional exposure to high sound pressure), a normal tympanogram, and a positive research stapedial reflex with a positive Metz test (≤60 dB).

Group B was composed of 20 subjects (10 male and 10 female; average age of 59.7 years, range 44–68, and standard deviation 12.9). They were observed after microvascular decompression of the trigeminal nerve in 4 cases, stereotactic radiosurgery for primary trigeminal neuralgia in 3 cases, and after radiosurgery and surgery of vestibular schwannoma in 13 cases in this group. Subjects with diabetes and hypertension were excluded. In all cases, the stapedial reflex was absent and the tympanogram was normal. Auditory brainstem response (ABR), performed during the diagnostic phase, was all abnormal: in 4 cases there was an absence of waves, in 16 cases there was a delay in V wave latency, and in 16 cases there were interaural latency differences between waves I–V.

### 2.2. Medical Treatment

Daily oral administration of 600 mg of ALA acid for 60 days. The subjects enrolled were given drug packaging and received a call from the principal investigator at least once a week. They could also contact the service through a dedicated telephone number if they had any further questions. All patients signed an informed consent form.

### 2.3. Audiological Evaluation and Questionnaire Survey

The study protocol was scheduled within two time points: T0, before starting therapy, and T1, after the end of therapy. The same evaluation protocol was applied in both examinations.

Pure-tone thresholds for air conduction were measured and reported as averages of four frequencies (0.5, 1, 2, and 4 kHz), indicated as 4FA, for the ear with tinnitus in the monaural case or the ear with more severe tinnitus in the bilateral case. Additionally, high-frequency PTA (4, 6, and 8 kHz), indicated as PTAhf, was computed for the same ear [49].

Psychoacoustic pitch (expressed in kHz) and loudness (expressed in dB) matches were determined for each subject.

Clinical audiometers and earphones (Interacoustic AC 40, by Interacoustic, Middle Fard, Denmark and Telephonics TDH 39 P, by Interacoustic, Middle Fard, Denmark) generated and reproduced the acoustic signals necessary for the task, respectively, in a double-walled sound-proof cabin.

Each patient was invited to adjust the frequency of a pure tone (presented at a comfortable level) to match the subjective pitch of their own tinnitus. The tone was presented to the ear contra-lateral to the side of the tinnitus or to the side with less intense tinnitus, if bilateral. The loudness match was tested by employing a bracketing approach with 1-dB increments. The subjective loudness level (SLL) of tinnitus was computed by subtracting the hearing threshold from the absolute loudness match. Three replications were performed, and the median value was calculated.

All patients were asked to complete the tinnitus handicap inventory (THI) [50], Italian version [51], to determine the overall degree of perceived annoyance at the beginning and at the end of therapy (T0–T1).

The answers to the 25 THI questions are in the form of “yes”, “sometimes” or “no”, with corresponding scores of 4 points, 2 points, or 0 points. This leads to a total score ranging from 0 to 100, which represents the worst annoyance. On the basis of the total score, the system provides five levels: level 1-non or slight handicap (THI 0–16), level 2-mild (THI 18–36), level 3-moderate (THI 38–56), level 4-severe (THI 58–76), level 5-catastrophic (THI 78–100).

The questionnaire consists of three groups of questions that identify three subscales: “functional”, “emotional” and “catastrophic”. The functional subscale reflects role limitations in the areas of mental, occupational, and physical functioning. The emotional subscale includes items representing a broad range of affective responses to tinnitus (anger, frustration, irritability, and depression). The catastrophic subscale reflects patients’ desperation, perception of having a terrible disease, and lack of control.

### 2.4. Statistics

A database has been implemented. To maintain patient confidentiality, spreadsheets submitted to the principal investigator were fully anonymous and did not include any identifiable data about patients or caregivers. A paired t-test was used to compare the audiometric parameters and the scores of the THI and its subscales before and after treatment. Statistical significance was set at *p* < 0.05 in all of the analyses for which the Statistical Package for the Social Sciences (SPSS) software version 26.0 was used.

The study was conducted in accordance with the Declaration of Helsinki and approved by the Institutional Review Board of Modena (protocol n. 2163, approved on 6 July 2010, registered on 7 July 2010). All patients signed an informed consent form.

## 3. Results

In Table 1, the audiometric parameters (4FA, PTAhf, pitch, and SLL) and THI scores of both groups at T0 are reported. As detected, there were no significant differences in parameters except for 4FA, which was statistically worse in group B.

**Figure 1:**
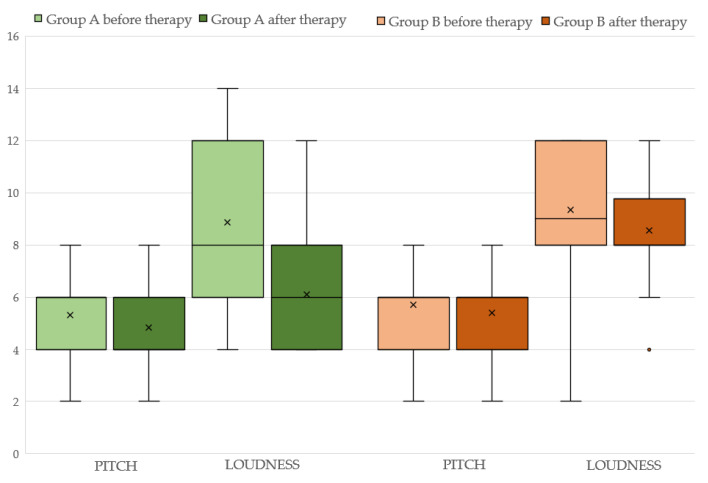
Pitch and loudness before and after therapy in Group A (light and dark green color respectively) and Group B (light and dark brown color).

**Figure 2:**
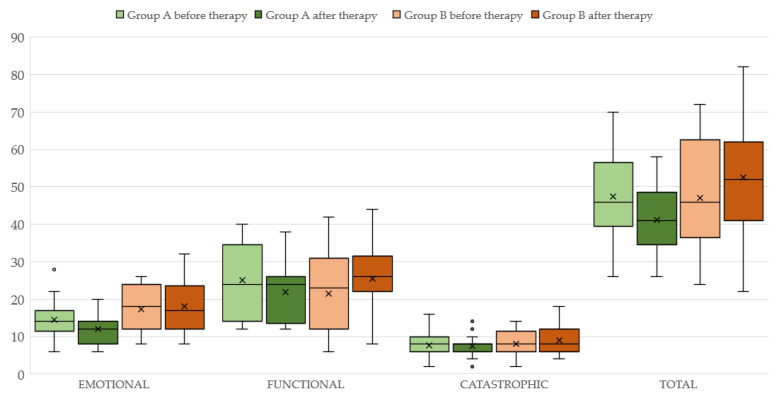
THI total score and its subscales before and after therapy in subjects of Group A (light and dark green color, respectively) and Group B (light and dark brown color).

Concerning group A, the aforementioned variables before and after the therapy with ALA are displayed in Table 2 (see also Figure 1). It can be seen that both 4FA and PTA hf improved after therapy (*p* < 0.05 and *p* < 0.0001, respectively). Additionally, the loudness parameter (SLL) is significantly reduced (*p* < 0.005) at the final assessment. Statistically significant differences are observed for both the “functional” (*p* < 0.05) and “emotional” (*p* < 0.005) subscales but not for the “catastrophic” subscale (*p* > 0.05) of the THI. The total THI score is significantly reduced (*p* < 0.005) after ALA treatment (see also Figure 2).

The results of therapy in group B are displayed in Table 3 (see also Figure 2). Neither 4FA nor PTAhf differ between the two assessments. (*p* < 0.05). The total THI score and its “functional”, “emotional” and “catastrophic” subscales scores before and after the therapy with ALA are also unchanged (*p* < 0.05).

## 4. Discussion

This study investigated the off-label use of the ALA R-enantiomer in the treatment of CTS by means of an internationally validated self-assessment measure (THI) and audiometric examinations. The use of self-reported measures to assess patients’ functional status has attracted interest because it is of great help in the approach to complex dysfunctions in which molecular and systemic alterations coexist. Moreover, there is still a lack of objective markers to evaluate the effects of therapies in the treatment of CTS. Tinnitus perception is correlated with emotional impact [52], and self-report questionnaires help in estimating the severity of tinnitus. Thereby, it is important to understand the perceptions of subjects concerning the effects of therapy in these clinical pictures.

A beneficial effect of ALA treatment was observed only in patients with bilateral cochlear dysfunction and metabolic syndrome. The improvement of tinnitus annoyance was supported by THI scores, but, interestingly, only the “emotional” and “functional” subscales of the THI were significantly reduced after therapy, while the “catastrophic” scale did not improve. There is no evident explanation for this discrepancy, possibly; in subjects with peripheral syndromes, central neural hyperactivity was more often triggered in response to cochlear damage [14]. Moreover, as the catastrophic scale reflects a feeling of having a terrible disease and a lack of control, which are more stable personality traits, a more extended follow-up would be necessary to appreciate the eventual improvement in this domain.

The mean improvement in the THI total score corresponds to the minimal clinically relevant change for evaluating treatment effects [53]. Since it was shown that THI significantly predicts depression, anxiety, and stress [54], we can conclude that a reduction in functional limitations is accompanied by the amelioration of distress, even though beneficial psychological knock-on effects deserve further investigation [55].

Both 4FA and PTAhf were significantly improved by ALA supplementation; these results partially resemble those of a large cross-sectional study that showed higher intakes of antioxidants were associated with better PTAs at both speech and high frequencies [56]. This effect can also be attributed to the antioxidant properties of ALA, which is highly reactive against a variety of ROS [57], as hyperglycemia and inflammation, important components of the metabolic syndrome, increase the production of these oxidant factors [58]. Moreover, ALA has intrinsic properties that facilitate glucose metabolism [40].

ALA bio-availability and plasma concentrations, although synthesized by the human body, are not enough to meet the energy requirements of the cells. ALA is mostly obtained from the diet [59], but supplementation seems to have beneficial effects on mitochondrial function and exhibits direct free radical scavenging properties [60]. In fact, mitochondrial DNA shows a mean common deletion in the cochlear tissue of patients with age-related hearing loss compared to controls [61]. Furthermore, cytochrome c oxidase subunit 3 expression, one of the three mitochondrial DNA-encoded subunits of respiratory complex IV, is significantly reduced in the spiral ganglion of patients with presbycusis in relation to their peers with normal hearing [62]. Since lipoic acid supplementation prevents the loss of cytochrome c oxidase activity, it hypothetically attenuates any putative decrease in cellular energy and redox status [63]. The data suggest that ALA has a short half-life and low bioavailability (about 30%), triggered mainly by its hepatic degradation and reduced solubility [40].

A recent study showed that ALA supplementation (at a slightly different dosage from our study) improves vascular tone and may have a beneficial effect on cardiovascular health in overweight/obese youths [64], indirectly indicating a positive effect on inner ear circulation.

A large study showed that dietary antioxidant intake is associated with a reduced prevalence of age-related hearing impairment (ARHI), but there was no temporal link between diet and the incidence of hearing loss in an older population [65]. This discrepancy might be due to several factors, such as the type, dose, and duration of dietary supplementation applied to hearing-impaired patients. On the other hand, a more recent study reported that nutritional interventions for obesity, including supplementation with (among others) alpha-lipoic acids, lecithin, tea, and ginseng, may protect against the development of ARHI [66].

It is uncertain whether cochlear dysfunction and the consequent hearing loss are the only suspected underlying causes of tinnitus in patients with metabolic syndrome since obesity and insulin resistance are deemed to be the major pathomechanisms of micro-vasculopathy in the central nervous system [67]. A recent randomized, double-blind, placebo-controlled clinical trial that excluded retrocochlear lesions suggested the efficacy of antioxidant supplementation with vitamins, minerals, and phytochemicals combined with ALA on tinnitus parameters and subjective discomfort [68]. In this way, antioxidant therapy seems to reduce subjective discomfort and tinnitus intensity in tinnitus patients.

Concerning our results from questionnaires, no great benefit in the treatment of tinnitus in subjects with auditory nerve dysfunctions was shown. That said, it is noteworthy that the PTAs at T0 in group B were more severe, and this aspect did not facilitate any benefit from ALA supplementation. THI scores suggest a benefit from this antioxidant compound in the cochlea, especially in the stria vascularis [69], which has been shown to play a critical role in hearing function. The cochlea, with its single arterial supply system (few collateral vessels), is considered highly sensitive to vascular changes, such as those promoted by diabetes mellitus, dyslipidemia, and hypertension.

The THI total score and its functional subscale in group B showed a trend toward statistical significance, but this does not mean that the *p*-value would have become more significant if more subjects had been enrolled and tested. On the contrary, it is likely that the *p*-values would become less significant if extra data were collected [70].

This study has some limitations, first of all the lack of a placebo group and the small number of subjects included. Moreover, no data concerning metabolic and oxidative stress markers to appreciate blood changes due to ALA supplementation are available, nor are there any data from a longer follow-up after therapy. Another limitation is due to the case studies that reported THI scores indicating a moderate level of annoyance in most cases.

## 5. Conclusions

Chronic subjective tinnitus is difficult to evaluate objectively, and there are no objective markers that represent the diagnosis or therapeutic effect of tinnitus. However, this study indicated that antioxidant therapy with the R-isomer form of ALA could contribute to reducing both the perceived intensity of the tinnitus and psychological distress in subjects with cochlear dysfunction associated with metabolic syndrome. On the contrary, no significant positive effect was obtained with regard to tinnitus annoyance in patients with auditory nerve dysfunctions. More appropriately designed clinical studies are needed both to understand the role of oxidative stress in the pathophysiology of functional disorders of the inner ear and to appreciate the role of antioxidants in the treatment of tinnitus-related auditory disorders.

## Figures and Tables

**Table 1:** Variables are expressed as means and standard deviations (SD). 4FA = pure tone average of the ear with tinnitus or more severe tinnitus if bilateral, PTAhf = pure tone average high frequencies, Pitch = frequency match between tinnitus and pure tone expressed in kHz, SLL (subjective loudness level) expressed in dB, THI total score and its subscales. Independent T-Test: *p* < 0.005 = (**); df = degree of freedom.

	Group A		Group B		Ind. T-Test		
	Mean	SD	Mean	SD	t	df	*p*
4FA	31.4	9.4	40.6	9.2	−3.80	48	<0.0001 **
PTAhf	54.4	12.5	48.9	6.9	1.78	48	0.053
Pitch	5.3	1.9	5.7	1.6	−0.75	48	0.455
Loudness (SLL)	8.9	2.9	9.3	2.7	−0.59	48	0.556
THI total	47.4	10.9	47.0	13.8	0.95	48	0.925
THI (functional)	25.1	9.7	21.5	10.6	1.24	48	0.220
THI (emotional)	14.5	4.9	17.4	6.8	−1.76	48	0.085
THI (catastrophic)	7.7	3.5	8.1	3.6	−0.36	48	0.723

**Table 2:** Audiometric parameters and THI scores before and after therapy in subjects of Group A.

	Before Therapy		After Therapy			Paired T-Test	
	Mean	SD	Mean	SD	t	df	*p*
4FA	31.4	9.1	30.1	9.4	−2.38	29	0.024 (*)
PTAhf	54.4	12.5	48.6	10.4	8.2	29	<0.0001 (**)
Pitch	5.3	1.9	4.8	1.6	1.2	29	0.240
Loudness (SLL)	8.9	2.9	6.1	2.0	4.37	29	<0.0001 (**)
THI total	47.4	10.9	41.3	9.4	4.4	29	<0.0001 (**)
THI (functional)	25.1	9.7	21.9	8.2	2.57	29	0.015 (*)
THI (emotional)	14.5	4.9	11.9	3.7	3.3	29	0.002 (**)
THI (catastrophic)	7.7	3.5	7.4	2.9	0.68	29	0.502

Variables are expressed as means and standard deviations (SD). 4FA = pure tone average of the ear with tinnitus or ear with more severe tinnitus if bilateral, PTAhf = pure tone average high frequencies, Pitch = frequency match between tinnitus and pure tone expressed in kHz, SLL expressed in dB, THI total score and of its subscales. Paired T-Test: *p* < 0.05 = (*), *p* < 0.005 = (**); df = degree of freedom.

**Table 3:** Audiometric parameters and THI scores before and after therapy in patients of Group B.

	Before Therapy		After Therapy		Paired T-Test		
	Mean	SD	Mean	SD	t	df	*p*
4FA	40.6	9.2	41.2	7.5	−0.721	19	0.480
PTA hf	49.9	6.9	49.6	6.9	−0.659	19	0.524
Pitch	5.7	1.6	5.4	1.6	1.37	19	0.186
Loudness (SLL)	9.3	2.7	8.5	2.0	1.22	19	0.237
THI total	47.0	13.8	52.5	14.5	−1.88	19	0.075
THI (functional)	21.5	10.7	25.5	9.7	−1.83	19	0.083
THI (emotional)	17.4	6.8	18.0	6.4	−0.448	19	0.659
THI (catastrophic)	8.1	3.6	9.0	3.6	−1.143	19	0.267

Variables are expressed as means and standard deviations (SD). 4FA = pure tone average of the ear with tinnitus or more severe tinnitus if bilateral, PTAhf = pure tone average high frequencies, Pitch = frequency match between tinnitus and pure tone expressed in KHz, Loudness expressed in dB, THI total score and of its subscales. Paired T-Test: *p* < 0.05; df = degree of freedom.

## Data Availability

The data presented in this study are available on request from the corresponding author due to privacy restrictions.
